# VQProtect: Lightweight Visual Quality Protection for Error-Prone Selectively Encrypted Video Streaming

**DOI:** 10.3390/e24060755

**Published:** 2022-05-26

**Authors:** Syeda Maria Gillani, Mamoona Naveed Asghar, Amna Shifa, Saima Abdullah, Nadia Kanwal, Martin Fleury

**Affiliations:** 1 Department of Computer Science, Faculty of Computing, The Islamia University of Bahawalpur, Bahawalpur 63100, Pakistan; maria.gillani@iub.edu.pk (S.M.G.); saima.abdullah@iub.edu.pk (S.A.); 2 School of Computer Science, College of Science and Engineering, National University of Ireland Galway, Co., H91 TK33 Galway, Ireland; 3 Department of Information Security, Faculty of Computing, The Islamia University of Bahawalpur, Bahawalpur 63100, Pakistan; amna.shifa@iub.edu.pk; 4 School of Computing and Mathematics, Keele University, Staffordshire ST5 5GB, UK; n.kanwal@keele.ac.uk; 5 Department of Computer Science, Lahore College for Women University, Lahore 54000, Pakistan; 6 School of Science, Technology and Engineering, University of Suffolk, Ipswich IP4 1QJ, UK; fleury.martin55@gmail.com

**Keywords:** confidentiality, channel errors, forward error correction, selective encryption, video quality assessment

## Abstract

Mobile multimedia communication requires considerable resources such as bandwidth and efficiency to support Quality-of-Service (QoS) and user Quality-of-Experience (QoE). To increase the available bandwidth, 5G network designers have incorporated Cognitive Radio (CR), which can adjust communication parameters according to the needs of an application. The transmission errors occur in wireless networks, which, without remedial action, will result in degraded video quality. Secure transmission is also a challenge for such channels. Therefore, this paper’s innovative scheme “VQProtect” focuses on the visual quality protection of compressed videos by detecting and correcting channel errors while at the same time maintaining video end-to-end confidentiality so that the content remains unwatchable. For the purpose, a two-round secure process is implemented on selected syntax elements of the compressed H.264/AVC bitstreams. To uphold the visual quality of data affected by channel errors, a computationally efficient Forward Error Correction (FEC) method using Random Linear Block coding (with complexity of O(k(n−1)) is implemented to correct the erroneous data bits, effectively eliminating the need for retransmission. Errors affecting an average of 7–10% of the video data bits were simulated with the Gilbert–Elliot model when experimental results demonstrated that 90% of the resulting channel errors were observed to be recoverable by correctly inferring the values of erroneous bits. The proposed solution’s effectiveness over selectively encrypted and error-prone video has been validated through a range of Video Quality Assessment (VQA) metrics.

## 1. Introduction

The substantial developments in 5G network technology and the cellular devices’ computing capabilities have made mobile multimedia communication appealing to users. As a result, standard video-streaming applications along with numerous multimodal multimedia applications, such as online gaming and immersive 360${}^{\circ }$ applications, have spread to such devices. Mobile multimedia communication requires substantial network resources, in terms of available bandwidth and low-latency transmission, to maintain a high Quality-of-Service (QoS), which will translate into good Quality-of-Experience (QoE) for the end-user [1]. Consequently, codecs play an important role in video streaming to accommodate the channel’s limited bandwidth and storage capacity. For example, the H.264/Advanced Video Coding (AVC) standard [2] remains a widely deployed codec in real-time video streaming applications.

Despite the presence of codecs, there remains competition for bandwidth, which essentially depends on access to a sufficient spectrum. Prior licensed spectrum usage is usually quite uneven and depends heavily on the specific wireless applications and their market penetration. Within a Cognitive Radio Network (CRN) [3], an uneven and underutilized licensed spectrum resource can be exploited to speculatively acquire bandwidth for multimedia applications. By employing CRNs, secondary users (SUs) can flexibly analyze their data rates, sense and access those temporarily vacant bands to satisfy the desired data rates for reliable communication. Compared to traditional wireless network users, SUs can adjust their application parameters according to the network conditions to fulfill the need of various multimedia applications for better QoE [4]. Because CRN channels are a desirable type of wireless network for multimedia transmission, this has led CR to be considered as a potential candidate to be incorporated into 5G cellular networks [5]. CR technology, whether part of a 5G network or operating as an independent CRN network, for multimedia communications opens up a new paradigm in which mobile devices receive or transmit streamed video content in a temporarily vacant licensed band in an opportunistic manner. However, along with positive features, it remains risky to multicast confidential multimedia content in this way due to the open nature of wireless channels [6].

In addition to the risk to video content confidentiality and sensitivity, during all wireless transmission, including over CRN channels, channel impairments will ineluctably cause changes to the signal characteristics during transmission. The most common causes are noise and competing traffic across the channel, leading to signal attenuation, cross-talk, electromagnetic intrusions, and bandwidth restrictions [7]. As a result, error detection and correction are the key challenges in communication systems. Generally, error control coding for video applications is classified into four different methods: (1) Retransmission, (2) Error resilience, (3) Error concealment, and (4) Forward Error Correction (FEC) [8]. FEC is especially used in real-time multimedia applications when real-time data are needed and when the delay caused by retransmission requests is not tolerable. Random-error FEC [9] can be carried out as Block coding and Convolution coding. Block codes work on the data of fixed-sized blocks, whereas convolution codes work on bitstreams of arbitrary size. These codes are mostly used for error correction in real-time applications. Turbo code and the Viterbi algorithm are examples of convolution coding. Linear and nonlinear codes depend on the relationship between parity bits and information bits [10].

Aside from the need for video content protection in the interests of multimedia providers, with the growing enforcement of privacy laws, privacy protection of transmitted data is of primary importance [11]. Encryption is a well-known technique to provide confidentiality to data transmitted over insecure channels. Encryption can be applied as naïve, for the hiding of full video content, or selective encryption, which limits encryption to key elements in the compressed bitstream [12]. Consequently, the following key questions arise:

**Q1:**  What will happen to the selectively encrypted video data after transmitting over wireless channels, which are prone to errors?
**Q2:**  Will it be possible to recover the multimedia content with an equivalent visual quality?
**Q3:**  Will the decryption of error-corrupted video still succeed at the receiver end, or will it fail?

Considering these research questions, the contributions of this paper are as follows:

1. This paper provides a novel and pioneer prototype (to the best of the authors’ knowledge) for protecting the video quality of selectively encrypted H.264/AVC compressed videos while transferring over erroneous wireless networks.
2. Selective Encryption (SE) using two-round secure process is applied to the selected syntax elements of an H.264/AVC CABAC encoder to achieve video privacy, and it maintains the video’s format compliancy and compression efficiency for effective channel bandwidth utilization.
3. The Gilbert–Elliot model is implemented for the simulation of an error-prone channel.
4. A Random Linear Block coding-based FEC mechanism is deployed on the encrypted H.264/AVC bitstreams for the recovery of bit-errors. The results are verified using various Video Quality Metrics and evaluation criteria.

The remainder of this paper is organized as follows. Section 2 describes related studies to this subject. Section 3 describes the details of the proposed scheme. Section 4 illustrates the promising visual results based on video-quality metrics for the tested videos. The limitations of the work to date, together with future work extensions, are also given in this section. Finally, Section 5 concludes the paper by summarizing how its research fits into the current technological environment.

## 2. Related Studies

Several techniques have been investigated in the literature to address video quality issues over wireless channels [13,14,15,16,17,18,19,20,21,22]. Generally, QoS or QoE [3] over multimedia communication channels can be categorized according to various metrics including: Bit Error Rate (BER), Packet Loss Ratio (PLR), Blocking Probability (BP), Collision Probability (CP), Dropping Probability (DP), Energy Efficiency (EE), and Spectrum Efficiency (SE).

In [23], the authors proposed a utility-based H.264/Scalable Video Coding (SVC) video streaming scheme to improve quality over a multi-channel Cognitive Radio Network. In [24], the authors examined the comparative effects of channel errors on selectively encrypted video bitstreams by two H.264 codec entropy coders Context Adaptive Binary Arithmetic Coding (CABAC) and Context Adaptive Variable Length Coding (CAVLC). They determined the combined effect of compression and encryption on the video quality and concluded that CAVLC is more susceptible to channel error. CABAC is being utilized by all advanced encoders; then, this finding [24] serves as a base to devise an advanced bit-error protection method for CABAC entropy coder of H.264/AVC (as considered in this paper). In [25], an extended Selective Encryption (SE) model for both H.264/AVC and HEVC encoded streams was introduced in which the regular mode of CABAC was used. Motion, texture, and structure encryption was applied to the video frames for the partial protection of video content. The performance of the proposed technique was assessed by taking the relative PSNR and SSIM. The HEVC codec increases the compression efficiency but has less error-resilient features [13] and is more sensitive to channel bit errors because of more encoded redundant content [14]. Thus, HEVC encoded video requires more efficient error concealment techniques.

Previously, instead of error concealment, Chen et al. [15] had proposed an error resilience coding scheme for H.264/AVC video coding, in which a group of Macroblock (MBs) are encoded with adaptive intra-refresh. In [16], a hybrid of Block Boundary Matching and Directional Interpolation Error Concealment (DIECA) methods was employed to preserve the quality of 3D H.264/MVC encoded video transmitted over an error-prone wireless communication network. In this work, for the lost frames, a Depth-Assisted Error Concealment (EIDD-DAEC) [17], Bayesian Kalman Filtering for Error Concealment (BKF-EC), depth-assisted motion vectors (MVs), and the disparity vectors (DVs) are exploited to estimate and recover the corrupted colored frames and achieve the better objective and subjective quality. In [18], an effective channel-modeling scheme was implemented using source rate control and adaptive playback techniques. Furthermore, for enhancing perceptual video quality over a Cognitive Radio Network (CRN), the authors formulated a content-aware Channel Quality Index (CQI) metric-based channel allocation scheme [19].

Error bursts frequently occur in wireless channels due to slow or fast fading. They are also potentially more damaging to compressed video streams compared to isolated errors due to the fragile nature of a highly compressed video bitstream. The Gilbert–Elliot (GE) [26,27] is extensively used to represent ‘bursty’ error patterns in transmission channels, allowing analysis of the transmission efficiency, such as how many errors an error-recovery scheme detects and corrects induced in its channel.

Prior work has also focused on different video error control techniques including source and channel rate control [20], adaptive playback control [18], and error concealment [21,22]. However, the status of the received signal can only be predetermined from the transmitter signal if an accurate communication channel is modeled [9]. An FEC is a channel coding technique that adds redundancy to transmitted data to achieve error control [28] by which the transmitter sends data along with the redundant information and the receiver distinguishes and identifies only the original data and requires the redundant bits only in the case of some bit error or data loss. Unlike Automatic Repeat Request (ARQ), when an error is detected within the transmitted data, FEC restores it without requesting re-transmission. Thus, when transmission delay matters, FEC may be a quicker and more suitable option to choose [9]. In FEC, redundant bits are added to the original bitstream by the FEC encoder [29]. Hamming codes, Reed–Solomon (RS) codes, Hadamard codes, BCH codes, Expander Codes, Golay codes, and Reed–Muller codes are various common methods for adding redundancies to data [30]. The receiver checks sent data, performs error detection, followed by error correction according to the type of code, and accepts data only if they match the specification.

The authors [31] analyzed several FEC approaches for error-free transmission of videos in varying network scenarios. RS encoding is applied at the packet, frame, and sub-Group-of-Pictures (GoP) level by considering various attributes of the videos. The decision tree algorithm is used to apply RS codes to the encoding parameters of videos to maintain the required QoE. However, applying FEC at the packet level increases the network overhead due to the redundancy added to each packet. The authors [32] suggested a systematic content-dependent FEC technique which uses RS codes on H.264/AVC encoded frames. Although the results exhibit smoother video quality and higher performance gain, the scheme increased the complexity of the FEC encoding method. Recently, Nunome [33] presented a joint model of Application Layer FEC (AL-FEC) and an error-concealment technique for H.264/AVC video to assess the subjective QoE and objective QoS of videos transmitted over a noisy channel. This method adopted RS coding and concluded that the efficiency of the code rate is not only dependent on the condition of the transmission channels but also on the contents. Then, in [34], an adaptive source FEC coding scheme was proposed for the mitigation of end-to-end distortion during real-time video messages, and both consecutive and sporadic video frame drops were minimized.

In [35], an Exclusive OR (XOR)-based Publicly Verifiable Secret Sharing (PVSS) scheme called GRGPVSS was proposed. An FEC coding scheme dubbed PATON was presented to address issues affecting the effective delivery of high-definition mobile videos such as high transmission rate, limited bandwidth, transmission errors, and throughput fluctuations [36]. The researchers [37] applied an FEC strategy by considering Un-Equal Protection (UEP). The proposed method achieved the same performance as that obtained from the expanding window strategy but with an improved computational cost. A coded caching scheme was proposed based on Combinatorial Structures [38]. These structures are called resolvable designs and can be applied in a natural pattern. The scheme was implementable for a wide range of parameters. Then, in [39], researchers applied FEC and Opportunistic Routing (OR) and improved the QoS. They also achieved a lower Symbol Error Rate (SER). Furthermore, the proposed strategy (QFEC-OR) was more effective in respect to 20 to 80 QoS perception parameters. In [40], the researchers proposed an FEC algorithm based on the lookup table architecture of distance Bit Error Rate (BER) at the Medium Access Control (MAC) layer of wireless networks. Furthermore, the authors of [41] proposed an error correction and detection technique for noisy images to provide error-free transmission by adding parity bits to the bitstream before transmission.

To assess the impact of error control schemes, the authors of [42] studied the performance of various quality metrics of error-concealed images and videos using multiple error-concealment techniques. They concluded that objective video quality is more appropriate to measure the performance of error recovery techniques. The authors of [43] proposed a novel Impulse Noise Detection and Mitigation (INDAM) technique. This method involves a complex Cyclic Redundancy Check (CRC) method, replacing the affected pixels by using surrounding pixels based on complex mathematical calculations.

However, the error method proposed in this paper is a bit-inversion technique, which, by detecting the errors in encoded bitstreams and simply inverting bit errors, results in an improved, reduced computational cost. It can be concluded from related studies that although many schemes have been proposed to improve the quality of videos, little work is currently being undertaken on video security and error recovery applied jointly, specifically for Multimedia CRNs. This paper proposes a pioneer mechanism (to the best of the authors’ knowledge) equipped with error correction for selectively encrypted compressed videos. The comparative analysis of the proposed scheme using different parameters is given in Table 1, which shows the clear distinction and research contribution of the proposed VQProtect over existing methods.

## 3. Proposed Solution

This paper proposes a joint protection and error recovery mechanism for compressed videos against transmission losses and unauthorized access to the content while preserving the video quality intact of the original video and utilizing a minimum of bandwidth. The proposed scheme is implemented in following three phases; the details are discussed in the subsequent subsections.

- In the *first phase*, video content is compressed and protected at the same time. The privacy protection is implemented using a two-round secure process. First, data diffusion is achieved by applying permutation on selected residuals data of compressed H.264/AVC bitstreams, and later, the XOR encryption algorithm is applied to the permuted data. The compressed selectively encrypted video bitstreams are produced as an output of this phase.
- In the *second phase*, channel modeling is performed through the Markov-Chain based Gilbert–Elliot model, which introduces bit errors inside the selectively encrypted videos (output of *Phase 1*) and enables simulations of the burst error effects of communications links.
- In the *last phase*, an FEC mechanism is applied (on both the encoder and decoder side) to detect and correct bit errors from the H.264/AVC selectively encrypted bitstreams (output of *Phase 2*) for their error-free transmission.

### 3.1. Compression and Privacy Protection

The two-round secure process is applied within the compression stage of a H.264/AVC encoder. Entropy coding is the last compression stage of the H.264/AVC hybrid encoder. There are multiple residual parameters output from a CABAC entropy coder [51] that are suitable for encryption without compromising its compression efficiency. These residuals are Transform Coefficients (TCs), Motion Vector Differences (MVDs), delta Quantization Parameters (dQPs), and the arithmetic signs of TCs and MVDs. For selective encryption, two-round operations are applied to the signs bits of MVDs and the TCs levels (color components) of the compressed bitstreams. The selective encryption on MVDs and TCs keeps the video format compliant (playable) and without escalating bit rates. As previously discussed in the research of [24], H.264/AVC’s CAVLC is more susceptible to channel errors as compared to CABAC. Thus, this paper devised a protection scheme for the H.264-CABAC entropy coder, which can be applied to HEVC compression, with a few comparatively minor modifications.

**Figure 1:**
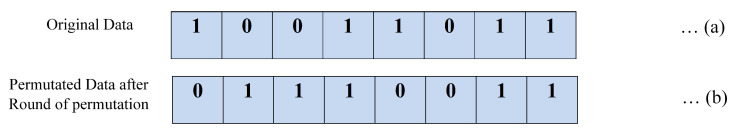
Bit permutation round: (**a**) actual bit positions, (**b**) bits are right-shifted circularly by three bit positions.

For computationally efficient encryption rounds, a simple XOR cipher is used, which, however, can be easily compromised. Therefore, a further layer of data diffusion is added with a permutation round on selected sign bits of Motion Vector Differences (MVDs) and Transform Coefficients (TCs) levels. It is important to notice that in the software implementation of CABAC encoder, sign bits are allocated a byte, so that within the software code, actually bits within sign bytes are permuted. In each byte of chosen syntax elements, the bits are right-shifted circularly by three bit positions, as shown in Figure 1. The number of all permutations obtained with eight-bit elements is “8!” so, it is equivalent to 40,320 permutations per byte operation are performed.

After applying a permutation round on selected bytes, XOR encryption is applied on permuted data bytes. An *n* bit encryption key is used, with at least 128 bits in length. Lower values of *n* increase the risk of a brute-force attack being applied, and some may prefer a 256-bit key for additional protection. Let, $C_{i}$, $K_{e}$, and $M_{g}$ denote the encrypted data, the encryption key, and the original message, respectively.

(1) $$C_{i}=\mathrm{Enc}\left(K_{e},M_{g}\right)=K_{e}⊕M_{g}$$

On the receiver side, the reverse process takes place, i.e.,

(2) $$M_{g}=\mathrm{Dec}\left(K_{e},C_{i}\right)=K_{e}⊕C_{i}$$

The ciphered data are then transmitted through a channel to the receiver, where it is again XORed with the key to decipher the original data before reverse permutation.

### 3.2. Channel Modeling

In this paper, the noisy channel is simulated with Gilbert–Elliot (GE) channel, which refers to a wide class of finite-state fading channels that model communication links with memory. This model is extensively simulated in research studies to find packet losses [52], frame losses [53], and bit losses [54], hence making it an appropriate choice for modeling the impact of errors within a noisy wireless channel [55].

The GE model is based on a discrete-time hidden Markov chain with two states that are the Good or Gap (*G*) state and the Bad or Burst (*B*) state. Let S={G,B} be the state space of the transmission channel. The probabilities of error occurrence in *G* and *B* states are considered as P(G) and P(B), respectively [56]. The GE model is computationally expensive when considering error events in both states. For the sake of simplicity and efficiency, we have implemented a fixed GE model by assuming the probability of errors in the *G* state is fixed. Accordingly, P(G)=0 represents that the event (error occurrence) in the *G* state will not happen, and all bits will be transferred correctly in the *G* state, while P(B)=1 represents that the event (error occurrence) in a *B* state will happen. For inducing a small number of error bits, we assume P(G)>P(B) that the *G* state has a higher probability compared to the probability of error occurrence in the *B* state. Along with the probability of error occurrence, the transition probability (probability of switching from one state to another state) is also considered, which are of two types, i.e., *self-transition* and *cross-transition probability*. In *self-transition probabilities*, $P_{GG}$ is the probability of a transition from *G* to *G* state again, while the probability of a transition from *B* to *B* state again is $P_{BB}$.

**Figure 2:**
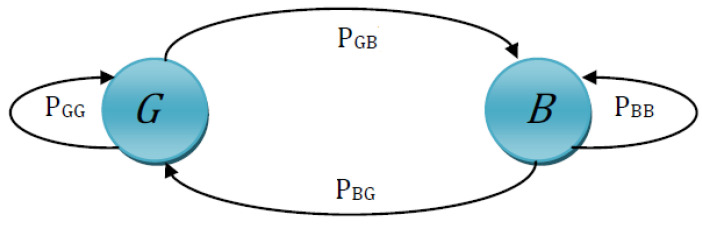
State Transitions of Gilbert–Elliot Channel Model.

In the *cross-transition probabilities*, $P_{GB}$ denotes the probability of some *G* state next entering the *B* state and $P_{BG}$ denotes the probability of some *B* state next entering the *G* state [57]. For example, when a current bit is received with an error (*B*) and the previous bit was transmitted correctly (*G*), the transition from *G* state to *B* state takes place and vice versa for *B* state to *G* state. The sum of all the probabilities for a particular state is always 1, such as $P_{BB}+P_{BG}=1$ and $P_{GG}+P_{GB}=1$. A state transition diagram of the GE channel model is given in Figure 2.

The channel states will evolve according to a Markov chain. The self-transition probabilities (3) and cross-transition probabilities (4) are computed as follows:

(3) $$\begin{array}{c} P_{BB}=1-P_{BG}\;\;\mathrm{and}\;\;P_{GG}=1-P_{GB} \end{array}$$

(4) $$\begin{array}{c} P_{BG}=1-P_{BB}\;\;\mathrm{and}\;\;P_{GB}=1-P_{GG} \end{array}$$

Moreover, GE Model uses a 2×2 transition matrix of two states *G* and *B*, at time *T*, to determine the state transition probabilities.

(5) $$M=\left[\begin{array}{cc} P_{GG} & P_{GB}\\ P_{BG} & P_{BB} \end{array}\right]$$

(6) $$M=\left[\begin{array}{cc} 1-P_{GB} & P_{GB}\\ P_{BG} & 1-P_{BG} \end{array}\right]$$

Furthermore, the stationary probabilities for the *G* and *B* states are denoted by $\pi_{G}$ and $\pi_{B}$ respectively. The stationary distribution of a Markov chain is a probability distribution that remains unchanged in the Markov chain as time progresses. Typically, it is represented as a row vector π whose entries are probabilities summing to 1 as $\pi_{G}+\pi_{B}=1$, and transition matrix $\Pi =M_{x}^{⊤}\times \Pi$ [58]. Now, $\pi_{G}=P_{GG}\times \pi_{G}+P_{BG}\times \pi_{B}$ and $\pi_{B}=P_{GB}\times \pi_{G}+P_{BB}\times \pi_{B}$, so finally, the stationary probabilities for the G and B state are given in (7).

(7) $$\pi_{G}=\frac{P_{BG}}{P_{BG}+P_{GB}}\;,\;\pi_{B}=\frac{P_{GB}}{P_{GB}+P_{BG}}$$

The mean sojourn time (the expected amount of time for which the channel remains in one state before moving to the other state (either in *G* or *B*)) of being in that state was also calculated. As in this work, the channel is assumed to be in one of the two states only, i.e., *G* or *B*, so the mean sojourn time of state *G* and state *B* denoted by $T_{G}$ and $T_{B}$, respectively, can be estimated through (3) and (4) as:

(8) $$T_{G}=\frac{1}{1-P_{GG}}\;,\;T_{B}=\frac{1}{1-P_{BB}}$$

After that, the steady-state probabilities (the probability of channel errors occurring in the steady state) were also calculated. The steady-state probability of being in state *G* or state *B* in terms of mean sojourn time is denoted by $P_{GG}$ and $P_{BB}$, respectively, and is calculated as [59]:

(9) $$P_{GG}=\frac{T_{G}}{T_{G}+T_{B}}\;,\;P_{BB}=\frac{T_{B}}{T_{B}+T_{G}}$$

In this paper, when the communication channel is in one of two states (say *G* or *B*), the Bit Error Rate (BER) at each time is calculated. Hence, the probability of being in a *G* or *B* state is dependent on the number of bit errors per unit time known as Mean Bit Error Rate ($M_{BER}$), such as $M_{BER}=\left(P_{GG}\times P_{g}\right)+\left(P_{BB}\times P_{b}\right)$, where $P_{g}$ is the probability of error occurrence in the *G* state and $P_{b}$ is the probability of error occurrence in state *B* [26]. In our implementation, the calculated $M_{BER}$ was varied from 0.07 to 0.1%, and it was computed over the total number of bits transmitted per sequence. For the implementation of the fixed GE model, $P_{g}$ was set to zero (0) and $P_{b}$ was set to 0.8. The probabilities of being in a good and bad state, $P_{GG}$ and $P_{BB}$, are dependent on $M_{BER}$ in the equation above. For further implementation details on channel error modeling upon selectively encrypted video, refer to the paper [24]. It should be noticed that the GE model is used to introduce bit errors in the selectively encrypted bitstream, assuming that the occurrence of bit errors is independent of each other. The implementation steps of the GE channel model are summarized below.
**GE
Channel Modeling Algorithm for Error Encoding****Step1:** Obtain the encrypted data or the encrypted and FEC encoded data to be sent over a communication channel.**Step 2:** Determine the state of the transmission channel, i.e., is it in a good or bad state?**Step 3:** Determine the stationary state probabilities $\pi_{G}$ for the good state and $\pi_{B}$ for the bad state.**Step 4:** Determine the sojourn time $T_{G}$ and $T_{B}$ of both states.**Step 5:** Determine the steady-state probabilities $P_{GG}$ and $P_{BB}$.**Step 6:** Calculate the mean Bit Error Rate.**Step 7:** Induce the errors according to the calculated mean BER (varied within 0.07 to 0.1%) in the *B* state only.**Step 8:** Forward the data with errors added toward the decryption and decoder modules.

### 3.3. Forward Error Correction

**Figure 3:**
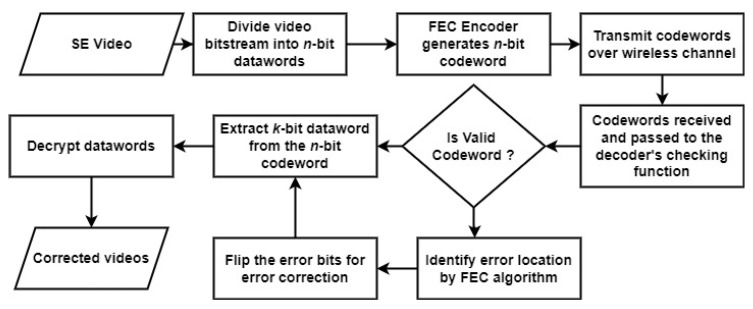
FEC method for improving the video quality when errors occur.

The magnitude and location of errors must be known before the correction process. FEC [29] is carried out with block coding that herein works on the macroblock level of a video data stream. In block coding, the data stream is divided into blocks of fixed size called a codeword. The flow chart in Figure 3 depicts the overall working of the FEC methodology to restore video quality in the face of transmission errors. The FEC code can be used to detect and correct both single bit and multibit errors to achieve sufficient QoE.

The input data stream consists of two segments, i.e., information bits with length ‘*k*’ (called the dataword) and some redundant bits of length ‘*r*’ (called the parity or check bits). These check bits are added to each dataword block to form a larger block of total length ‘*n*’, which is the codeword. Redundancies can be added either to the start, at the end of the frame, or somewhere in between. Hence, Codeword=Dataword+paritybits, which is expressed in linear form as n=k+r.

**Figure 4:**
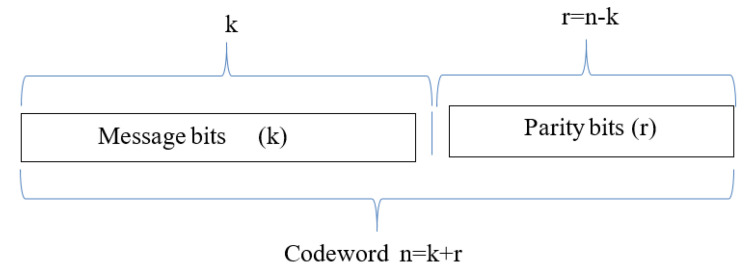
An (n,k) block code.

Thus, $2^{k}$ possible different datawords can be created with *k* bits. Similarly, $2^{n}$ possible different codewords can be created with *n* bits. Codewords contain more bits than datawords due to the additional check bits, i.e., n>k, from which it follows that $2^{n}>2^{k}$. Hence, $2^{n}$ codewords can possibly arise when a codeword is decoded. Out of these $2^{n}$ codewords, only $2^{k}$ codewords are valid, and the remaining $2^{n}-2^{k}$ codewords are invalid. Thus, for (n,k) block codes, $2^{k}$ codewords have a regular structure (valid) and the remaining $2^{n}-2^{k}$ have invalid bits, as illustrated in Figure 4. If the codeword is received as an invalid codeword, an error has occurred.

The (n,k) code used in this study for experiments is a (7,4) linear block code, where n=7, k=4, so r=n−k=3. A block code is said to be a linear code if it satisfies the following three conditions: (i) the all zero word is always a codeword i.e., 00000000, (ii) given three codewords, i.e., $C_{x}$, $C_{y}$, $C_{z}$, these must satisfy the condition $C_{z}=C_{x}+C_{y}$ and then $d\left(C_{x},C_{y}\right)=w\left(C_{z}\right)$, and (iii) the minimum distance of the code must be equivalent to the minimum weight of that codeword, i.e., $d_{\min}=w_{\min}$. Moreover, a small block is chosen for the FEC as larger block sizes result in longer reconstruction delays at the receiver [44].

In the implemented FEC, matrices are used for codeword generation rather than generating codewords manually. Thus, for (n,k) codes, *L* is the generator matrix consisting of *k* rows and *n* columns, given in (10).

(10) $$L=\left[\begin{array}{cccc} l_{11} & l_{12} & \| & l_{1n}\\ l_{21} & l_{22} & \| & l_{2n}\\ \vdots & \vdots & \; & \vdots \\ l_{k1} & l_{k2} & \| & l_{kn} \end{array}\right]$$

where *L* is constructed by combining the identity matrix and the parity-bits matrix as:

(11) $$\begin{array}{cc} \; & L_{k\times n}=I_{k\times k}|P_{k\times (n-k)}\\ \; & L_{k\times n}=I_{k\times k}|P_{k\times r} \end{array}$$

where *I* is the (k×k) identity matrix, and *P* is the (k×(n−k)) or (k×r) matrix for (n,k) codes. The generator matrix *L* in our case, i.e., for (7,4) block codes, is given as:

(12) $$L=\left[\begin{array}{ccccccc} 1 & 0 & 0 & 0 & 1 & 0 & 1\\ 0 & 1 & 0 & 0 & 1 & 1 & 1\\ 0 & 0 & 1 & 0 & 1 & 1 & 0\\ 0 & 0 & 0 & 1 & 0 & 1 & 0 \end{array}\right]$$

For (n,k) codes, the codewords in a linear block code can be expressed in vector form by multiplying the generator matrix with the data block of information bits to be transmitted as C=F×G. Here, the input information bits or decoded *k* bits are denoted by *F* and expressed in vector form as $F=[F_{1},F_{2},F_{3},\ldots ,F_{k}]$. Then, these codeword bits or encoded *n* bits sent over transmission channel are denoted by ‘*C*’, and can be expressed in vector form as $C=[C_{1},C_{2},C_{3},\ldots ,C_{k-1},C_{k},C_{k+1},\ldots ,$$C_{n-1},C_{n}]$, and C=F×G. These are the codewords received by the receiver after being passed through the noisy/error-prone channel. Thus, there is also a possibility of errors in the received codewords. The errors may be denoted by *E*, and here, there may be up to *n* errors in a number, which can be represented in vector form as $E=[E_{1},E_{2},E_{3},\ldots ,E_{n}]$, where $E_{j}=1$ represents “error at the jth position” and $E_{j}=0$ means “no error”. If $C^{\prime }$ is the received codeword, then in vector form, $C^{\prime }=\left[C_{1}^{\prime },C_{2}^{\prime },C_{3}^{\prime },\ldots ,C_{n}^{\prime }\right]$. Then, $C^{\prime }=C+E$

(13) $$\begin{array}{c} C^{\prime }=\left(C_{1},C_{2},C_{3},\ldots ,C_{n}\right)+\left(E_{1},E_{2},E_{3},\ldots ,E_{n}\right) \end{array}$$

(14) $$C^{\prime }=\left(\begin{array}{c} \left.(C_{1}+E_{1}\right),\left(C_{2}+E_{2}\right),\\ \left(C_{3}+E_{3}\right),\ldots ,\left(C_{n}+E_{n}\right) \end{array}\right)$$

For a single received word, the word will be calculated as $C^{\prime }=C+E$. The parity check matrix is constructed using a generator matrix at the decoder side. For (n,k) codes, *D* is the generator matrix consisting of r=(n−k) rows and *n* columns.

(15) $$D=\left[\begin{array}{cccc} d_{11} & d_{12} & \ldots & d_{1n}\\ d_{21} & d_{22} & \ldots & d_{2n}\\ \vdots & \vdots & \; & \vdots \\ d_{r1} & d_{r2} & \ldots & d_{rn} \end{array}\right]$$

where *D* is constructed by combining a parity bits matrix and identity matrix in such a way that $D_{(n-k)\times n}=P_{(n-k)\times k}^{T}|I_{\left(n-k)\times (n-k\right)}$ or $D_{r\times n}=P_{r\times k}^{T}|I_{r\times r}$ where transpose $P^{T}$ is the ((n−k)xk) or (rxk) matrix and *I* is the identity matrix of ((n−k)x(n−k)) or (rxr) for (n,k) codes. Thus, the parity check matrix *D* for (7,4) block codes and the transpose of the parity check matrix, i.e., $D^{T}$ will be

$$D=\left[\begin{array}{ccccccc} 1 & 1 & 1 & 0 & 1 & 0 & 0\\ 0 & 1 & 1 & 1 & 0 & 1 & 0\\ 1 & 1 & 0 & 1 & 0 & 0 & 1 \end{array}\right]\;,\;D^{T}=\left[\begin{array}{ccc} 1 & 0 & 1\\ 1 & 1 & 1\\ 1 & 1 & 0\\ 0 & 1 & 1\\ 1 & 0 & 0\\ 0 & 1 & 0\\ 0 & 0 & 1 \end{array}\right]$$

Let *S* be the error syndrome (where S=0 or S≠0), which is computed as $S=C^{\prime }\left(D^{T}\right)$. When the result of the syndrome is zero, i.e., S=0, then no error has been detected and vice versa. Hence, the error bits can be corrected by altering their values from 0 to 1 or from 1 to 0 with non-zero syndrome detection. If the number of errors is beyond the code’s error-correcting capacity but within the detection capability, the syndrome is said to indicate uncorrectable errors. The proposed code is designed in such a way that there is a one-to-one correspondence with an individual error of an *E* vector for each combination of syndrome bits. Thus, error correction can easily be achieved. It is also noticeable that the syndrome is purely a function of the error patterns and not the actual or transmitted codewords. For a single received word, it will be $C^{\prime }\times D^{T}=E\times D^{T}$. This states that the received codeword entirely depends on the error pattern *E* if some codeword has been affected by errors. Suppose that there are no errors; then, $E\times D^{T}$ will have a zero value, indicating the absence of errors. Otherwise, $E\times D^{T}$ will result in a non-zero value indicating the presence of one or more errors. Finally, if the received codeword matches any valid codeword, the dataword is extracted by removing the parity bits to obtain the original data. Otherwise, an error(s) is detected and corrected. The pseudocode of the implemented FEC on the sender and receiver sides is given in Algorithm 1 and 2.
**Algorithm 1** Pseudocode of Implemented FEC Method: Source/Sender1:Construct a generator matrix $L_{k\times n}$ by using identity matrix $I_{k}$ and parity matrix $P_{k\times r}$ as L=[I|P], where k=4, n=7 and r=32:**while** End of Data **do**3:    Extract the four encrypted information bits i.e., $F_{x},F_{x+1},F_{x+2},F_{x+3},F_{x+4}$4:    Multiply *F* with *L*, such that:5:    $C_{1}=F_{1}\times \left[\begin{array}{c} l_{11}\\ l_{21}\\ \ldots \\ l_{k1} \end{array}\right]$, $C_{2}=F_{2}\times \left[\begin{array}{c} l_{12}\\ l_{22}\\ \ldots \\ l_{k2} \end{array}\right],\ldots$, $C_{n}=F_{n}\times \left[\begin{array}{c} l_{1n}\\ g_{2n}\\ \ldots \\ l_{kn} \end{array}\right]$6:    Construct $C=(C_{1},C_{2},C_{3},\ldots ,C_{n})$7:    Send codeword *C* over the noisy channel8:    Move the value of *x* to next position9:**end while**

**Algorithm 2** Pseudocode of Implemented FEC Method: Destination/Receiver Side
1:Construct a parity check matrix $P_{r\times n}$ by using parity matrix $P_{r\times k}$ and identity matrix $I_{r}$ as D=[P|I], where r=3, n=7 and k=42:Take the transpose of Matrix *D* as $D_{n\times r}^{T}$, i.e $D_{7\times 3}^{T}$3:**while** End of Codewords being received **do**4:    Take erroneous codeword $C^{\prime }$5:    Multiply $C^{\prime }$ with $D^{T}$, such that6:    $S_{1}=C^{\prime }\times \left[\begin{array}{c} d_{11}\\ d_{12}\\ \ldots \\ d_{1n} \end{array}\right]$, $S_{2}=C^{\prime }\times \left[\begin{array}{c} d_{21}\\ d_{22}\\ \ldots \\ d_{2n} \end{array}\right],\ldots$, $S_{n}=C^{\prime }\times \left[\begin{array}{c} d_{r1}\\ d_{r2}\\ \ldots \\ d_{m} \end{array}\right]$7:    Calculate the Syndrome $S=(S_{1},S_{2},\ldots ,S_{n})$, i.e., in (7,4) block codes $S=(S_{1},S_{2},S_{3})$8:    **if** $S=0|S_{1}=0$ AND $S_{2}=0$ AND $S_{3}=0$ **then**9:        PRINT “No Error in the codeword”10:    **else**11:        Find error pattern *E* against Syndrome *S* in the Syndrome Table.12:        Calculate $C=C^{\prime }+E$ // Erroneous bit will be altered via this operation13:    **end if**14:    Extract the information bits from the codeword15:    Forward it to Decryptor16:
**end while**



## 4. Experimental Results and Performance Evaluation

Experiments were carried out on an Intel Core 2 Duo Processor with 4 GB RAM and a 64-bit operating system. The GE model and FEC mechanism were both simulated in the C++ programming language. The JSVM encoder (version 9.19) was used for the compression of videos as a single-layer H.264/AVC bitstream. The Main/High profile was chosen, as the Baseline profile of JSVM does not support CABAC encoding. The Intra and Inter frames (I+P+B) were encoded and selectively encrypted in a bitstream generated for a Group of Pictures (GOP) size and Intra period, which was equal to 16 and with a chroma subsampling pattern of 4:2:0. Experiments were performed at different Quantization Parameter (QP) values (i.e., 8, 12, 24, 36, and 48). The proposed scheme was tested on ten (10) test videos having different color pixels, motion activity, texture, and resolution. The results are taken on different resolutions, such as Common Intermediate Format (CIF) (352 × 288 pixels/frame), 4CIF (704 × 480 pixels/frame), and High Definition (HD) (1280 × 720 pixels/frame) with different frame rates (CIF at 30 fps, while 4CIF and HD at 60 fps). All test videos are publicly available at Derf’s collection [60].

**Figure 5:**
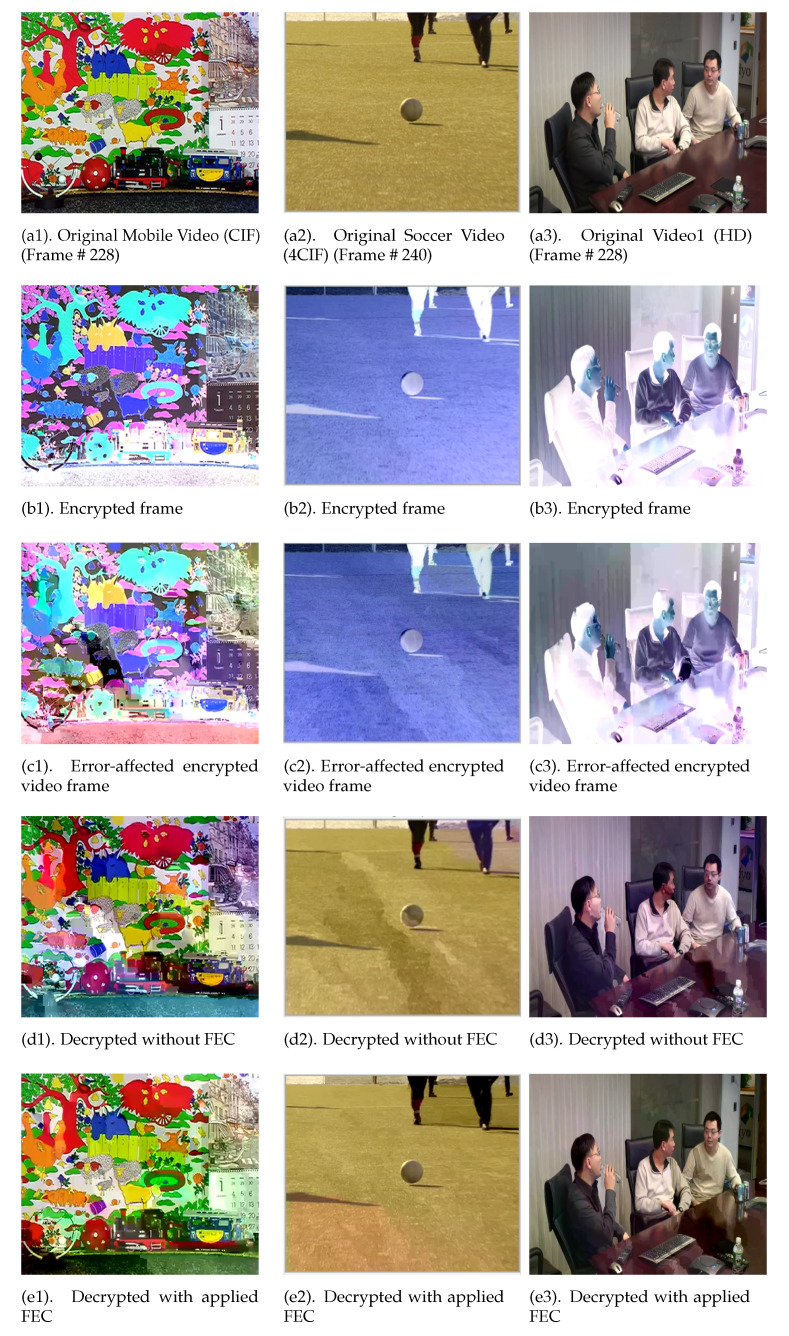
Visual representation of the effect of the implemented FEC scheme on different resolution test videos (**a1**–**a3**) Original video frames, (**b1**–**b3**) Selectively encrypted video frame, (**c1**–**c3**) Error-affected encrypted video frame, (**d1**–**d3**) Decrypted video frame without applying FEC and (**e1**–**e3**) Decrypted video frame with FEC applied.

The visual results and subjective quality of the videos are shown in Figure 5, which exhibits the implemented scheme’s effect on tested CIF, 4CIF, and HD videos at various stages. Figure 5 (d1, d2, d3) shows the decoded form of videos given in Figure 5 (c1, c2, c3), respectively, where a significant degradation in the visual quality can be observed. Figure 5 (e1, e2, e3) presents the decoded videos after recovering by using FEC, where video quality improvement can be visually observed.

**Figure 6:**
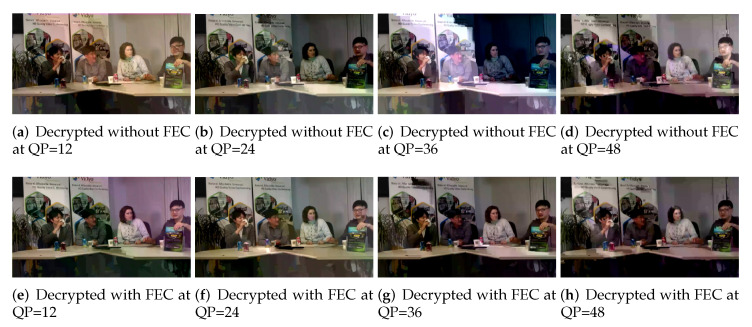
Effect of FEC at QP 12, 24, 36, and 48 on the Four People (HD) video.

Experiments were also performed at five QP values (8, 12, 24, 36, and 48). QP, which is reciprocal to the video quality, is also a representation of the quality of perception, with lower QPs representing higher quality video from a range for H.264/AVC and HEVC from 0 to 51. Figure 6 shows the comparative effect of FEC at different QP values (12, 24, 36 and 48) on decrypted videos with and without FEC. The impact of PSNR at different QPs on test videos is summarized in Table 1. The visual results show that an overall increase in visual quality after applying FEC, particularly at QP 24.

### 4.1. Video Quality Analysis

The selectively encrypted test videos with channel errors (induced using the GE channel model) were evaluated before and after the application of FEC. The quantitative evaluation employed various video quality metrics such as Peak Signal to Noise Ratio (PSNR) [61], Structural Similarity Index Measure (SSIM) [62], Mean Squared Error (MSE) [63], and Video Quality Model (VQM) [64]. In addition to the assessment of blurring and blocking effects [65], histogram analysis is also performed on the original and recovered videos.

#### 4.1.1. PSNR

PSNR [57] is used for quality estimation in terms of the reconstruction of ‘lossy’ compression and the noise (error) introduced in the signal (original data) during transmission. It is calculated as $\mathrm{PSNR}=10\times \log_{10}\frac{{\left(2^{n}-1\right)}^{2}}{\mathrm{MSE}}$, where $\left(2^{n}-1\right)$ is the maximum pixel value of the image (e.g., 255 for 8-bit representation) and MSE is the Mean Squared Error value (described in Section 4.1.3). A higher PSNR value (in decibels (dB)) indicates a better quality video. Generally, a PSNR that is between 20 and 25 dB is considered to be acceptable for wireless transmission quality loss [66].

The PSNR values given in Table 1 show an increase in PNSR values in all Y, U, and V color components in the recovered videos when decoded with the proposed addition of FEC compared to the PSNR of the decoded videos without error correction, which suffer from noise. Moreover, results were evaluated for five different QPs (8, 12, 24, 36, and 48) to further determine the FEC’s effect. The PSNR of the decrypted video sequences at different QPs, with and without FEC, is summarized in Table 2. The results show that at a QP value of 8, the visual quality in terms of the chrominance components is better. An apparent increase in Y-PSNR (Y indicates the luminance component) is observed at QP 24. This shows that the overall increase in visual quality is best at QP 24 (Figure 6f), as the human visual system is more sensitive to luminance than chrominance (recorded in the U and V components).

#### 4.1.2. SSIM

**Figure 7:**
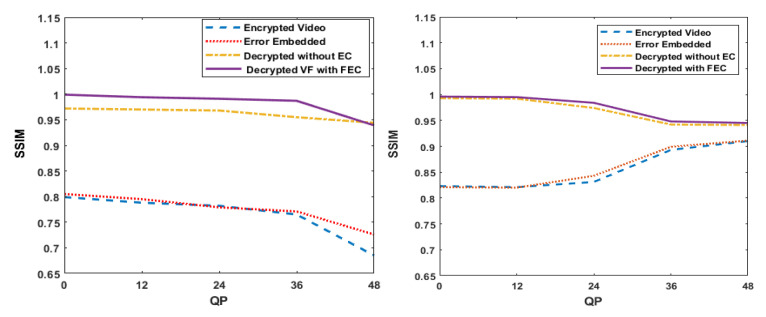
Comparison of average SSIM at different QP values (8, 12, 24, 36, and 48) for (**a**) Crew and (**b**) Soccer video (EC: error correction and VF: video frame).

SSIM calculates the similarity of the two images using structural distortions to evaluate the perceptual distortion based on the difference in the luminance values as given in Equation (16). It ranges from 0 to 1. It can be observed in Figure 7 and Table 2 that the SSIM plots of decrypted videos with FEC are better than the respective videos decrypted and decoded without FEC at their corresponding QPs, and it is lower for the QP value of 48, even after applying FEC. Table 2 depicts that the SSIM values are not always consistent between two videos of similar resolution.

(16) $$\mathrm{SSIM}=\frac{\left(2\mu_{x}\mu_{y}+C1\right)\left(2\sigma_{xy}+C2\right)}{\left(\mu_{x}^{2}+\mu_{y}^{2}+C1\right)\left(\sigma_{x}+\sigma_{y}+C2\right)}$$

where μ (mu) represents the mean luminance contrast, whereas σ denotes the standard deviation for variance contrast of two *x* and *y* frames being compared. The C1 and C2 are the constant to ensure function stability when the denominator becomes zero.

#### 4.1.3. MSE

**Figure 8:**
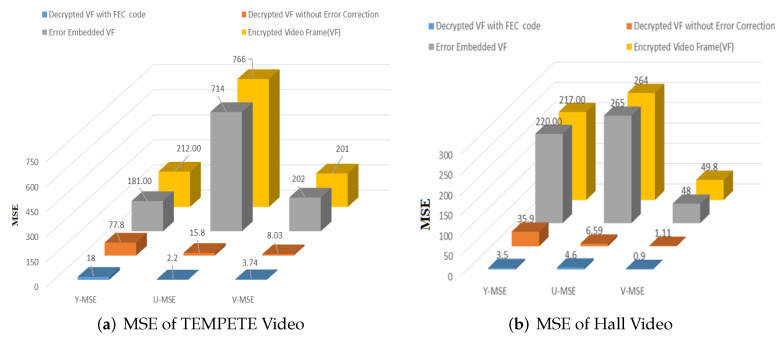
Comparison of MSE for (**a**) Tempete and (**b**) Hall videos (VF: video frame).

MSE is compared across each of the three channels in an RGB image and is the average of the squared differences between the luminance (Y) and chrominance (U, V) values of corresponding pixels in two frames (the decoded frame and the original frame). It may range from 0 to 65025, but a smaller value indicates a resulting better video quality. Table 3 and Figure 8 show the comparative MSE values at various phases of the proposed scheme. Figure 8 shows the MSE of luminance and chrominance, i.e., Y, U, and V. It is apparent that the average MSE of video recovered with FEC presented in Table 3 and Figure 8 led to the same conclusions as for PSNR and SSIM measurement. MSE is directly related to PSNR, as can be observed by the equation $\mathrm{MSE}=\frac{\sum_{i=1,j=1}^{m,n}{\left(Y_{i,j}-X_{i,j}\right)}^{2}}{mn}$, where *m*, *n* represents the width and height of the video frame, respectively, while *X* is the original frame and *Y* denotes the reconstructed frame.

#### 4.1.4. VQM

**Figure 9:**
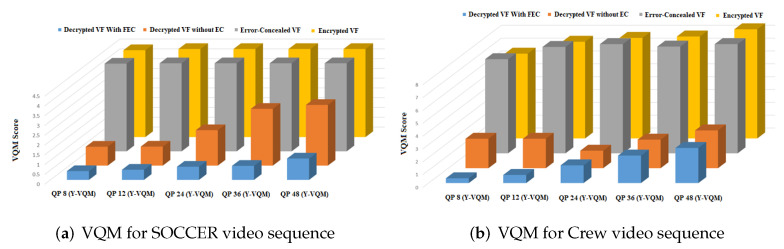
Comparison of VQM variation at different QP values (8, 12, 24, 36, and 48) of (**a**) Soccer and (**b**) Crew videos (EC: error correction and VF: video frame).

VQM is a reduced-reference quality metric in which seven parameters related to the video quality are extracted and linearly combined to estimate the VQM quality score. Thus, it includes a combination of objective parameters to evaluate the perceptual effects of an extensive range of distortions. The value of VQM may vary from 0 to *∞*. Zero VQM means no difference between a particular video and the original one. The higher the VQM value, then the higher the difference between the original video and the video under consideration. It can be observed from Table 3 and Figure 9 that videos decrypted with FEC code have minimal values of average VQM, confirming that there are minimal differences with the original videos. Figure 9 also demonstrates that the value of VQM increases with the increase in QP value.

#### 4.1.5. Histogram Analysis

**Figure 10:**
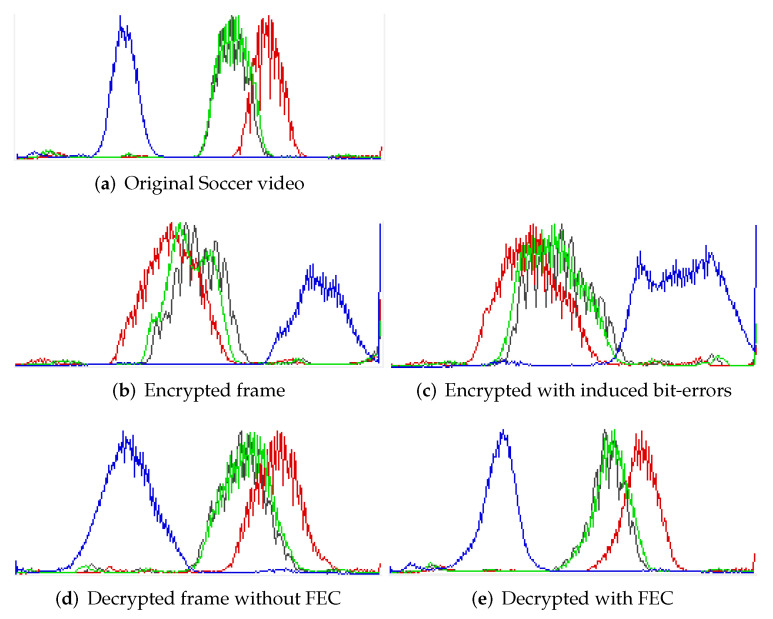
Histogram analysis of error-embedded video recovered without and with applying FEC on soccer video frame (4CIF) (frame no. 240).

A histogram can show the frequency distribution of color component pixel values (red, green, and blue components) before and after encryption and correction of errors. A histogram also determines the correlation of sample video frames with the original frames, before processing by encryption, and the application of FEC. A low correlation represents a more significant variance and vice versa. Figure 10 shows the SOCCER video (frame no. 240) histogram for different encoding modes, considering that the decryption is performed with or without FEC over encrypted video. Figure 10e shows the distribution of color values (R, G, B) of the video recovered (decrypted), when after application of FEC, is nearly similar to the original frame (Figure 10a). This shows that using FEC achieves a worthwhile improvement in visual quality after transmission across an error-prone communication channel (Figure 10d vs. e).

### 4.2. No-Reference Video Quality Assessment

**Figure 11:**
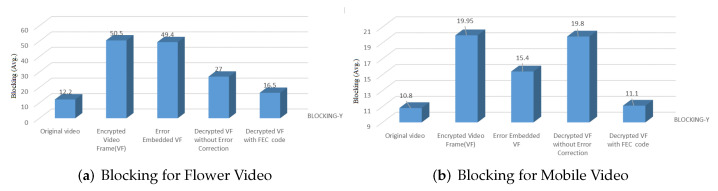
Comparison for Blocking for Error-embedded and recovered videos without and with FEC (VF: video frames).

To further validate the adopted FEC’s performance, the no-reference video quality metrics were computed to estimate the blocking and blurriness. The blocking problem occurs along the horizontal and vertical edges of a regular blocking grid. That grid in turn arises from the block-based processing of video codecs, including H.264/AVC. In contrast, blurriness is caused by the removal of high-frequency content from the original video/image signal. One way that may occur is if the quantization of frequency transform coefficients reduces high-frequency components to zero. Blurring is calculated by estimating the color variance in a pixel’s neighborhood after which the average variance is computed. Higher values indicate more significant blurring within transmitted video frames. The blurring and blocking metrics for test videos decoded with FEC are compared with the original videos, as shown in Table 4 and Figure 11, respectively. It can be observed from Table 4 that there is a slight difference between the original video and the decrypted video after recovery with FEC. In contrast, the video in other modes has a little more amount of blurriness. The results exhibit that coarse quantization results in increased values of both blurring and blocking.

### 4.3. Computational Cost Analysis

In our proposed scheme, the complexity of the encoded *n* bits of matrix *L* which have the dimension of (n−k) and *n*, sent over the transmission channel, is at most (n−1), whereas the complexity of the codewords received by the receiver after being passed through the noisy/error-prone channel is k of (n,k) codes. Hence, asymptotically, the worst-case complexity of applied FEC is O(k(n−1)). In comparison with our scheme, in [49], the authors achieved the reduced linear encoding complexity for LT codes of complexity $O(N_{s}$$log(N_{s}))$ but at the cost of performance loss. The complexity given in [48] is dependent on the number of iterations involved, GoP size and redundant packet allocation for P frames; however, in this method, the packet loss pattern may affect unlike levels of distortion and drop to an average value in the larger number of simulations, which would not happen in our scheme, as we are implementing FEC on codewords, not on individual frames.

**Figure 12:**
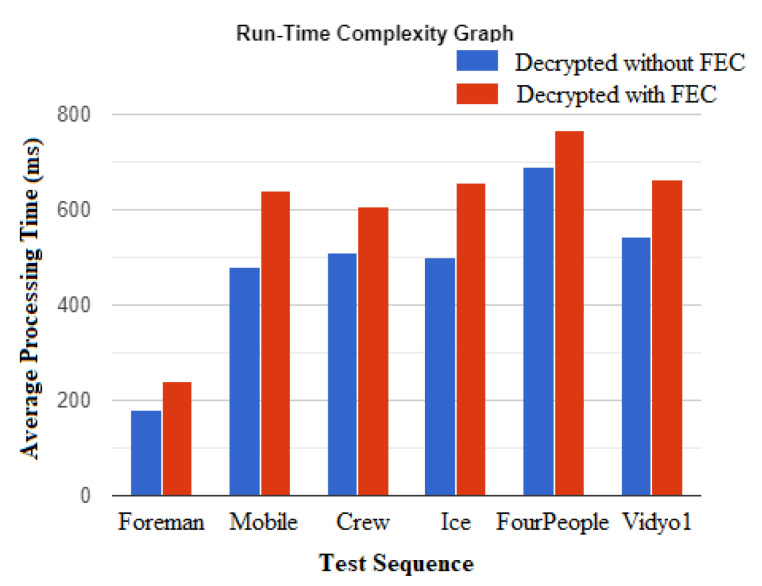
Comparison of average processing time for recovering videos without and with applying FEC.

The execution time was calculated for all the test videos to evaluate the run-time performance with and without FEC. Figure 12 shows the average computational time of recovered test sequences after timings were repeated multiple times. Figure 12 establishes that the average processing time (ms) of reconstructed video with FEC (214, 620, 600, 627, 787, 670) is a little bit higher than reconstructing videos without FEC (186, 491, 502, 500, 697) for test videos i.e., Foreman, Mobile, Crew, Ice, FourPeople, and Video1, respectively. However, the difference in time (ms) appears insignificant, producing values of 28, 123, 98, 127, 90, and 122, respectively, for all test videos and the comparative analysis given in Table 1, which also implies that the cost–benefit trade-off from applying FEC is much in favor of its application for error-prone videos.

### 4.4. Comparative Analysis

The proposed scheme can help protect video content both against unauthorized access and transmission errors while maintaining the video quality similar to that of the original video. Table 5 is a PNSR-based quantitative comparison of the proposed scheme with: state-of-the-art error correction by STBMA [67]; frame copy concealment by JM (JM-FC) [68]; and other recently proposed approaches [69]. Detailed results (see Table 5 column 6) show that the proposed scheme outperformed other techniques over all test videos. As can be seen, the PSNR difference between the original Intact video sequence, which was then H.264/AVC encoded at QPs of 22 and 32, and other methods of reconstructing the original video after errors had been introduced demonstrate that the proposed scheme (decrypted with FEC) achieved sufficient quality improvement.

## 5. Limitations and Future Work

In this paper, FEC is used to recover the bit errors that occurred during transmission to eliminate the need for retransmitting data, which, if retransmission is not needed, makes broadcasting, video streaming, and real-time applications more efficient. The work utilized Random Linear Block Codes to detect and correct errors with a single-bit error handling strategy. However, other methods for error recovery using convolution codes for burst errors can be designed in the future. In the future also, the implemented FEC method can be tuned to adjust the redundancy rate by predicting the loss rate or loss probability of the communication channel. One can adapt the redundancy rate via these predictions, which may allow lower bandwidth utilization if the predictions allow that. Given that a fixed redundancy overhead is a major compromising factor when using FEC, effective prediction of the loss rate would be particularly advantageous.

Cognitive Radio (CR) works by sensing the ambient wireless environment for a possibly temporarily vacant licensed bandwidth or by consulting online spectrum databases or by a combination of both methods of accessing additional bandwidth resources. In this paper, CRN channels are taken as an example, while channel error simulation is performed using the Gilbert–Elliot model. In the future also, the proposed scheme can be deployed over an experimental wireless testbed, which will allow comparative experiments, the results of which can be compared with software network simulation. Software network simulation can also be extended to encompass a variety of typical multimedia network setups.

To avoid complex encryption processing, combined bit permutation and XORing techniques were used. In the future, for testing the performance of the implemented FEC method, complex ciphers such as Chacha20 [70] and Advanced Encryption Standard (AES) [12] can be implemented as points of comparison.

For experimentation in this paper, the authors selected the H.264/AVC standardized codec because of its suitability to a wide range of interactive and non-interactive applications over various network configurations. In addition, the codec brings relative ease of software deployment as well as a range of supported low-cost hardware (e.g., Raspberry Pi) for rapid compression and decompression. On the other hand, both H.264/AVC and H.265/High-Efficiency Video Coding (HEVC) codecs use the same structures to stream and store video. However, the encoding process of HEVC and VP9 is more complex than the H.264/AVC coding standard in terms of execution time, CPU consumption, and hardware implementation [71]. Another good reason of using H.264/AVC is its support for various error-resilience schemes, allowing convenient assessment of their effect in H.264/AVC on QoE and QoS, should the need arise. On the other hand, H.265/HEVC and the currently emerging Versatile Video Coding (VVC) codec both lack some error resiliency techniques such as Redundant Slice and Flexible Macroblock Ordering (FMO). Hence, H.264/AVC [72] is more suitable for error protection other than through error concealment. In the future, it may be used over other advanced codecs such as High-Efficiency Video Coding (HEVC) [73], Google VP9 [74], and Versatile Video Coding (VVC) [75]. In fact, H.264/AVC’s CABAC entropy already exists in lightly modified form as the sole entropy coder of HEVC. This research can also further be expanded to the scalable coding environment, where the number of redundant or parity bits added will be dependent on the configuration of a scalable video bitstream.

## 6. Conclusions

During data transmission over 5G wireless networks incorporating CR, errors may propagate because of unwanted noise within the communication channels that may flip the bits of transmitted data from 0 to 1 or from 1 to 0, or it can be corrupted due to various other network artifacts, all of which can harm the video content. In today’s insecure environments, encryption is vital, but it can make the recovery of the original video data all the more difficult because of the corruption due to transmission and network errors. Considering these transmission scenarios of compressed videos, this paper proposed an effective scheme for protecting the visual quality of selectively encrypted compressed videos by utilizing the block coding-based FEC method. The FEC method corrects corrupted bits after locating them within blocks of the encrypted video bitstream. For securing the videos, selected syntax elements output by the CABAC entropy coder of H.264/AVC were encrypted by a two-round secure process, which permits real-time operation by low-complexity processing devices, such as those now being deployed within sensor networks. The visual and VQA results (Section 4) from applying the FEC method were compared with error-corrupted and selectively encrypted H.264/AVC videos, which were encoded and decoded without FEC. In the experiments with different QP values, the applied FEC algorithm’s performance was proven to be best at QP 24. Moreover, in the proposed method, the packet loss pattern may not affect unlike levels of distortion, as FEC is implemented on codewords, not on the individual frames. The complexity of our applied FEC is O(k(n−1)). The calculated execution time (Figure 12) shows the nominal increase in time (ms) when videos were encoded with FEC. The comparative analysis given in Table 1 also implies that the cost–benefit trade-off from applying FEC is much in favor of its application for error-prone videos. In the quantitative comparison, the proposed VQProtect also outperformed other techniques (Section 4.4 and Table 1) in terms of PSNR evaluation. The computational cost analysis and worst-case complexity of the implemented FEC scheme is a relatively simple method that does not noticeably increase computational complexity because it does not involve complex mathematical operations. Furthermore, it does not require any back-channel to re-transmit missing data in the event of persistent errors, causing transmission delay. Therefore, combining an FEC scheme with the selective encryption of compressed video streams is a way forward, especially for the anticipated incorporation of Cognitive Radio into the 5G radio initiative to increase mobile densification.

## Figures and Tables

**Table 1:** Analysis Comparing Existing Work with the Proposed Scheme.

Proposed Schemes	Video Format or Video Codec	Permutation applied				Video Quality Assessment						Analytical complexity	FEC Computational Average Time (ms)
			Encryption	Simulation Model	FEC	PSNR	SSIM	MSE	VQM	Blocking	Blurring		
[31]	H.264/AVC	X	X	No fixed loss rate	Reed–Solomon	X	Yes	X	X	X	X	RC Block Size Dependent	Not mentioned
[36]	HD Video	X	X	Monte Carlo	Systematic RS Block Erasure Code	Yes	Yes	X	X	X	X	O(M ($n_{max}-k_{1}+RM$))	87.2, 73.5, 62.3, 51, 40.3, 32.5, 24.5 (in different feedback frequencies)
[39]	Not given	X	X	WLAN	Reed–Solomon	No but SINR provided						Not mentioned	Not mentioned
[44]	Not given	X	X	GE Model	Reed–Solomon	No, but delay and redundancy provided						O($klog_{2}k+nlog\left(n\right)$)	70, 125, and 150 (for FEC-16, FEC-64, and FEC-128)
[45]	H.264/SVC	X	X	Monte Carlo	Recursive Systematic Cumulative Code	Yes	X	X	X	X	X	Low-complexity Table-look-up Operations Dependent	Not mentioned
[46]	IPTV data	X	AES	WLAN loss rate = 0.1	Systematic RS Block erasure code	No, but exposure rate and recovery probability provided						Not mentioned	Not mentioned
[47]	HFR video encoded with H.264	X	X	Monte Carlo	Systematic RS Block Erasure Code	Yes	X	X	X	X	X	O((M−1).R)	[0–2.5], [0–4.5] (for different frame rates)
[48]	H.264/AVC	X	X	Monte Carlo on AWGN channel	Luby Transform and Rate-Compatible Punctured Convolutional (RCPC) Codes	Yes	X	X	X	X	X	O($N_{s}log\left(N_{s}\right)$)	Not mentioned
[49]	HFR video encoded with H.264	X	X	Adaptive White Gaussian Noise (AWGN) Channel	Linear Block Codes	No, but Bit Error Rates and latency given						$O(N_{i}.K\left(n-k\right))$	Not mentioned
[50]	H.264/AVC	X	X	AWGN and Rayleigh Channels	Rate Compatible Punctured Convolution (RCPC) Codes	Yes	X	X	X	X	X	O(n)	Not mentioned
**VQProtect**	H.264/AVC	One Round	XOR algorithm	Gilbert–Elliot Model	Random Linear Block Codes	Yes	Yes	Yes	Yes	Yes	Yes	O(k(n−1))	35, 132, 109, 133, 99, 128 (for encrypted CIF, 4CIF and HD videos)

**Table 2:** Comparative PSNR at Five Different QP Values for Tested Videos.

		Average PSNR											
Encoding Mode	QP												
		**Crew**			**Soccer**			**Vidyo1**			**FourPeople**		
		**Y**	**U**	**V**	**Y**	**U**	**V**	**Y**	**U**	**V**	**Y**	**U**	**V**
	8	15.9	26.1	22.3	23.2	17.5	26.2	5.5	22	27.6	5.4	21.7	25.6
	12	16.1	24	20.2	23.5	17.8	26	5.8	22.0	26.1	5.9	21.1	24.8
Encrypted Video	24	16.5	23.9	20.5	24.7	17.9	25.8	6.3	22.1	26.9	6.1	21.6	25.3
	36	16.2	23.2	20.1	23.2	17.6	25.6	5.9	22.5	26.7	5.8	21.0	24.9
	48	14.9	21.9	19.8	23.1	17.1	25.5	5.6	22.4	26.3	6.0	21.4	25.5
	8	15.2	27.1	23	22.7	17.6	25.9	5.5	22.8	26.9	5.8	21.9	26.3
	12	16.8	25.4	20.8	23.1	17.4	25.5	5.6	22.0	26.7	5.8	21.2	25.6
Encrypted Videos with Errors	24	17.8	24.2	21	25.2	17.3	25.3	5.9	22.2	26.6	6.0	20.8	25.4
	36	16.2	23.8	20.5	24.9	17	25.1	5.7	23	26.9	5.9	20.9	25.4
	48	14.6	23.3	20	23	16.8	25.3	5.7	22.3	26.8	5.6	21.1	25.9
	8	29.8	36	39.4	35.7	53.3	53.4	21.9	37.9	38.9	17.2	34.7	35.9
	12	32.8	36	39.4	36.5	50.7	53	21.6	37.6	38.8	17.2	34.5	33.2
Decrypted Video without FEC	24	35.7	33.4	38.9	36.7	49.8	52.8	22.3	34.5	32.9	17.9	33.5	35.8
	36	30.5	32.2	37.1	35.1	47.1	52.5	18.6	33.5	33.9	16.5	33.6	33.4
	48	27.2	31.6	36.4	29	46.3	49.6	17.3	34.9	33.4	15.6	30.2	32.1
	8	36.8	49.3	50.4	38.9	60.5	54.3	20.1	40.1	40.5	18.1	37.2	38.4
	12	34.4	48.4	45.2	41.8	60.4	53.2	22.8	39.4	39.9	18.4	34.7	37.7
Decrypted Video with FEC	24	43.5	46	42.1	41.9	52.3	52.4	23.4	34.8	33.7	19.1	36.5	36.7
	36	44.3	37.1	39.5	42	48.4	52.1	18.2	34.6	33.9	17.6	35.3	36.6
	48	29.8	34.6	38.1	35.6	46.7	48.5	17.0	35.2	33.7	16.2	31.5	32.3

**Table 3:** Quality Metrics for Videos after Successive Processing Phases with and without FEC.

Phase	Metrics	Videos					
	(Avg.)	Flower	Hall	Tempete	Mobile	Four People	Vidyo1
	SSIM	0.84	0.78	0.84	0.82	0.05	0.03
Encrypted Video	MSE	212	217	212	114	15,457	16,813
	VQM	9.64	10.7	9.64	7.82	23.1	22.24
	SSIM	0.85	0.83	0.85	0.85	0.056	0.05
Encrypted Video with Errors	MSE	181	220	181	123	15,168	16,440
	VQM	8.07	9.8	8.07	7.87	22.7	22.0
	SSIM	0.96	0.97	0.96	0.93	0.84	0.87
Decrypted Video without FEC	MSE	77.8	35.9	77.8	21.5	592.1	357.7
	VQM	2.38	1.37	2.38	4.04	6.94	5.21
	SSIM	0.99	0.98	0.99	0.98	0.97	0.96
Decrypted Video with FEC	MSE	18	3.5	18	1.4	491.4	311.3
	VQM	0.79	0.89	0.79	2.3	5.98	4.80

**Table 4:** Comparison of Blurring for Test Videos.

Videos	Blurring (Average)			
	**Flower**	**Tempete**	**Mobile**	**Hall**
Original	[Y:4.64,U:0.17,V:0.15]	[Y:5.95,U:1.69,V:0.90]	[Y:8.48,U:2.21,V:2.07]	[Y:4.25,U:0.71,V:0.39]
Encrypted	[Y:10.3,U:1.51,V:1.26]	[Y:6.22,U:1.89,V:0.88]	[Y:9.89,U:2.61,V:2.31]	[Y:6.1,U:0.86,V:0.52]
Encrypted with errors	[Y:10.4,U:1.59,V:1.34]	[Y:6.52,U:2.07,V:0.86]	[Y:9.94,U:2.76,V:2.39]	[Y:5.05,U:0.89,V:0.53]
Decrypted without FEC	[Y:6.8,U:1.03,V:1.33]	[Y:6.19,U:2.09,V:0.88]	[Y:8.82,U:2.53,V:2.33]	[Y:4.96,U:0.79,V:0.43]
Decrypted with FEC	[Y:5.96,U:0.19,V:0.17]	[Y:6.07,U:1.73,V:0.89]	[Y:8.52,U:2.28,V:2.19]	[Y:4.53,U:0.74,V:0.41]

**Table 5:** Average PSNR Comparison of Proposed Method and Other Approaches.

Error-Coding Technique	QP	Average PSNR (dB)			
		**Foreman (CIF)**	**Crew (4CIF)**	**Ice (4CIF)**	**Average PSNR Difference (dB) from Intact**
Intact	22	41.35	41.78	43.70	-
	32	34.67	35.69	39.00	
JM-FC	22	37.60	39.21	39.18	3.61
	32	33.70	34.96	36.50	1.40
STBMA	22	39.49	40.64	41.74	1.65
	32	34.19	35.44	38.15	0.52
NDBV	22	39.99	39.03	40.58	2.41
	32	33.93	35.23	37.51	0.89
**VQProtect**	22	40.02	41.19	42. 29	1.14
	32	34.78	35.52	38.68	0.28
